# A Novel Approach for a Sustainability Evaluation of Developing System Interchange: The Case Study of the Sheikhfazolah-Yadegar Interchange, Tehran, Iran

**DOI:** 10.3390/ijerph17020435

**Published:** 2020-01-09

**Authors:** Aydin Shishegaran, Arshia Shishegaran, Gabriella Mazzulla, Carmen Forciniti

**Affiliations:** 1 Department of Water and Environmental Engineering, School of Civil Engineering, Iran University of Science and Technology, Tehran 16846-1114, Iran; aydin_shishegaran@civileng.iust.ac.ir; 2 Transportation Engineering Faculty, Islamic Azad University Central Tehran Branch, Pounak square, Tehran 19877-45815, Iran; Shishegaranarshia@gmail.com; 3 Department of Civil Engineering, University of Calabria, 87036 Rende, Italy; carmen.forciniti@unical.it

**Keywords:** sustainable development analysis, transportation infrastructure, traffic simulation, technique for order of preference by similarity to ideal solution (TOPSIS), applied effect of changes intensity in each indicator (AECIEI)

## Abstract

To resolve environmental and social problems and traffic congestion, a sustainability evaluation of transportation infrastructure should be done for current and proposed situations. The aim of the present investigation is to evaluate the sustainability of infrastructure project on system interchange and assess their results with respect to sustainable development indicators. Based on interviews with experts, intensive observation, and development infrastructure related to the case study, six proposed scenarios are proposed to improve traffic condition of the Sheikhfazolah-Yadegar interchange in Tehran. The proposed scenarios are modeled and calibrated in AIMSUN 8.0, then the value of all indicators are determined through an indirect and direct procedure by AIMSUN results. A technique for order of preference by similarity to ideal solution (TOPSIS) method is used to integrate the results of proposed indicators. Although the weight of each indicator specifies its effect in sustainability evaluation, changes intensity of indicator value was not applied in sustainability evaluation by the previous methods of multi-criteria decision analysis (MCDA). In this study, for the first time, a simplified approach is defined and used to evaluate sustainable development of all scenarios that can apply the effect of changes intensity in sustainability evaluation. This method is named the applied effect of changes intensity in each indicator (AECIEI). According to the purposes of Tehran vision in 2025, all proposed scenarios are ranked based on TOPSIS results and the simplified approach, and then results of these methods are compared.

## 1. Introduction

Transportation system is largely recognized having significant externalities affecting environment, society, and economy. Several externalities positively affect people lifestyle, in terms of economic growth, increase in educational opportunities and in land value too; transportation opportunities enhance the quality of life by providing mobility and physical access to resources, goods, markets, and other amenities [1,2]. Conversely, negative externalities affect in terms of air and noise pollution, energy consumption, traffic congestion, cost of living, and traffic crashes with direct negative consequences on public health, welfare, and people wellbeing. These negative aspects are particularly clear in mega cities characterized by a growth rate of population.

As a consequence, incorporating into transport planning actions inspired to sustainable development principles is of fundamental importance [3]. The economic development models adopted in the past decades were oriented to the economic growth only, without any regard to social or environmental impacts. However, more recently, the alternative paradigm named “sustainable development” was introduced as the development that “meets the needs of the present without compromising the ability of future generations to meet their own needs” [4]. A sustainable transport planning approach is aimed to provide accessibility as well as to enhance the quality of life through the regeneration of public space, in contrast with the traditional traffic-oriented approach focused on maximizing network capacity, traffic volumes, and travel speed [5]. More specifically, the sustainable development of highways and transport infrastructures can be defined by the selected option of construction, which can decrease environmental effects and improve the social and economic situation for the present and future generations [6,7].

Sustainable transport planning entails a constant monitoring action [3]; in this perspective, sustainable transport indicators play a vital role [8], and can help the decision-makers to choose among options or alternative scenarios.

Sustainable development content involves environmental, economic, and social indicators [9,10,11]. Indicators of sustainable transportation network (STN) were specified by the center of sustainable transportation (CST) by considering the following requirements [12]:

- Building and developing effective, economical, and adaptive transport infrastructure and transport system for all people who live in the world;
- Providing an environmentally friendly atmosphere, reducing waste, reducing consumed non-renewable resources, decreasing land use, decreasing noise pollution, and minimizing of using fossil fuels.

Today, eco-friendly modes of transport are used due to the harmful effects of transportation activities [13]; however, the critical point is how sustainability is going to be executed [14]. In addition, since the mid-1990s, the World Bank already claimed its will of funding only projects sustainable in economic, environmental, and social terms [15,16]. In this perspective, it is important to assess how transport system alternative solutions are compatible with the environment, as conventional evaluation methods are unable to predict it [17]. Some studies have been carried out in recent years, where the sustainable transport parameters in environmental, social, and economic indicators have been used to select the best option from a sustainable development point-of-view [13,18,19,20,21,22,23]. However, fewer studies focused on system interchanges connecting more highways, especially in a crowded population area with an increasing transportation demand as Tehran is.

The aim of this paper is to apply a multi-criteria decision analysis (MCDA) for evaluating the sustainability of an infrastructure project on system interchange where an original simplified approach is defined and used to compare alternative scenarios by applying the effect of changes intensity in sustainability evaluation. This method is named applied effect of changes intensity in each indicator (AECIEI). Indicators in the sustainable development definition were divided into environmental, social, and economic factors that are relevant to both the operational and construction steps. To calculate the weight of indicators, interviews with experts are carried out.

In the following, a brief theoretical background is introduced in order to highlight the contribution of the present research to the global knowledge. Section 3 presents the methodology and data used in the present work. This study involves four steps, including building scenarios by interviewing with experts, choosing eminent sustainable indicators and interviewing with experts for calculating a weight of indicators, accessing scenarios by evaluating sustainable development indicators, ranking scenarios, and choosing the great scenario. Finally, all of them are carried out in Section 4 and Section 5. The summary of the results is presented in Section 6 where AECIEI is compared with TOPSIS, and a discussion about the generalization of the methodology to other contexts is made.

## 2. Theoretical Background

Traditional transportation performance metrics tend to focus on vehicle mobility and congestion, neglecting the impact of transport on environmental, social, and economic sustainability.

Measuring and assessing different parameters of the sustainability of transportation performance and infrastructures is difficult. Combing different parameters into a single index is normally applied to compare and assess different paradigms. If developed properly, an indicator framework is an essential part of an effective transport planning process for internal management and decision support and for external communication [8].

In addition, the sustainability of the transportation concept should cover all scales and scopes over the long-term, taking into account the changes occurred in the indicators. The analyses inevitably grow in complexity [16]. The research in this field aims to develop methodologies able to faithfully reproduce the real situation and its evolution.

In the literature, several approaches have been suggested for evaluating the sustainability of transportation. The sustainability rating systems applied for transportation attempts to integrate sustainability in the decision-making process for transport infrastructure projects by using sustainability measurement indicators [24,25]. Many tools and methodological frameworks are available, among them rating systems, traditional decision-making techniques, checklists, and different evaluation frameworks and models. While these tools are highly valuable, there is also a need for more standardized tools to appraise the sustainability of transport projects [6].

Moving towards transport sustainability requires the constant monitoring and intensive evaluation of the current conditions through broadly accepted methodological tools such as indicators. The review proposed by Sdoukopoulos et al. [3] conducted an in-depth analysis based on the indicators included in the 78 initiatives. This analysis contributes to the development of a comprehensive picture of the evaluation process towards transport sustainability and aims at illustrating interesting findings on a number of relevant aspects. As a result, the indicators involved in the considered works can be grouped in the three main sustainability pillars (society, environment, and economy).

Most of the evaluations of transport sustainability are defined in large-scale. Zheng et al. [19] stated that the goals for transportation sustainability could best be achieved having appropriate metrics to determine what problems exist, where they exist, and consequently, what actions need to be taken. This research proposed an index of transportation sustainability as a tool that could be systematically applied for assessing the sustainability of places. The index comprises elements of the environmental domain, of the social domain and of the economic domain. Jeon et al. [22] compared 15 options for transportation and land use in Atlanta in 2013. They evaluated the sustainable development index by MCDA, in order to identify the most suitable plan for the predetermined sustainability-oriented objectives. As a limitation, the analysis does not explicitly capture the interactions among system performance, the economy, the environment, and social equity. Reisi et al. [26] developed a procedure for getting an index for local areas in Melbourne, by applying normalization, weighting, and aggregation methods. The composite indicator includes environmental, social, and economic sub-indices, and could help for better understanding of the measures and activities that influence the sustainability of urban transport. According to the importance of each indicator, a weight of indicator is specified in this research and many previous researches.

The novelty of some studies is categorizing into five topics: source usage; transportation; energy; water, and waste [27]. The rating system was used in the transportation system in a study proposed by McVoy et al. [28]. Transportation sustainability rating systems (TSRSs) depend on their workability. This approach is imperfect, and some disadvantages were reported when dealing with the concept of sustainability. Environmentally issues are preferred rather than operational issues. In this method, the criteria and weight selection are not clear [29]. Although this method is implemented at construction phases and planning, the European Union does not apply this method to implement decision-making analysis. MCDA and cost-benefit analysis (CBA) are used by EU members to make decisions, and TSRSs are not utilized to find the best option among various options by EU members [30].

There are many works where some possible transportation scenarios in a study region were evaluated with respect to environmental, social, and economic indicators. To improve traffic performance and decrease air pollution in the urban region, the sustainable development scenarios are proposed and applied for transportation infrastructures in the previous investigation. It seems that these works improved slightly in the urban region and improved traffic condition in larger region. In order to enrich the literature, the present work focuses on Sheikhfazolah-Yadegar interchange located in the urban area of Tehran (Iran).

An interchange is a road junction that has typically one or more ramps. The interchange is a useful infrastructure, which permits traffic on a different highway can transfer to others through the junction without interruption from other crossing traffic streams. The interchanges can connect three, four, and more ways. Two-level, four-way interchanges include Cloverleaf interchange, Stack interchange, turbine interchange, Windmill interchange, Roundabout interchange, and Hybrid interchange such as Cloverstack interchange [31,32,33]. The current system interchange of Sheikhfazolah-Yadegar is a Cloverleaf interchange, which should be used in low populated areas [34,35,36]. According to transportation demand in this region, changing system interchange can improve traffic condition.

In this study, seven scenarios (the current and six developed transportation infrastructures) recommended by experts are modeled to evaluate Sheikhfazolah-Yadegar interchange in Tehran. There are several useful studies that used MCDA method to select the best option in deciding sustainability evaluation and sustainable infrastructure [37,38,39,40,41]. In addition, several useful studies about traffic simulation and modeling traffic have been published in recent years [42,43].

In the present investigation, all scenarios are simulated in Trans CAD 5.0 software (Caliper Corporation, Newton, MA, USA), which can measure the influence area (IA) of these selected scenarios. All scenarios are modeled and validated in AIMSUN 8.0 (Aimsun SLU, Barcelona, Spain), and ten chosen indicators are calculated from AIMSUN results in an indirect and direct procedure. Based on the previous investigations, indicators in the sustainable development definition were divided into environmental, social, and economic factors that are relevant to both the operational and construction steps. To calculate the weight of indicators, interviews with experts are carried out, which are also used in evaluating and finding the development sustainable scenarios. In addition, the technique for the order of preference by similarity to ideal solution (TOPSIS) method is used to combine the effects of these indicators, which are stood on their significance with the experts’ point-of-view and the aims of Tehran insight in 2025. Therefore, TOPSIS method is utilized to evaluate and compare all scenarios in this study. In addition, a simplified approach, which is defined as the applied effect of changes intensity in each indicator (AECIEI), is used to a sustainability evaluation in the present study. The effect of changes intensity of each indicator is applied in this method. In the previous methods such as the analytic hierarchy process (AHP) and TOPSIS, just weight of each indicator is applied for summing values of all indicators of a scenario, and effect of changes intensity of each indicator is not applied. To apply the effect of changes intensity, AECIEI is defined and used in this study for the first time. Therefore, the first novelty of this study is referred to defining and using AECIEI. Comparing the results of TOPSIS and AECIEI is the second innovation of this study. The sustainable development evaluation on the system interchange is the third novelty of the present investigation.

## 3. Method and Data

### 3.1. Method

The aim of this study was the sustainability evaluation of current and developed transportation infrastructures, which were presented by different scenarios in this investigation. This evaluation was divided into five phases, described in the following.

(1) Modeling scenarios and measuring IA. According to the utilized source and interviewing with experts, all feasible scenarios that can improve the traffic situation in Sheikhfazolah-Yadegar interchange of Tehran were modeled in Trans CAD 5 software to measure their IA as the study region.
(2) Choosing eminent sustainable indicators and interviewing with experts to calculate a weight of indicators. Sustainability evaluation of transportation infrastructure plans was carried out with respect to environmental, social, and economic parameters. To assess each of these parameters, the relevant criteria to these parameters were defined to determine the sustainable scenarios. According to defined indicators and accessible data, ten indexes were selected including three sustainability principle. To calculate the weight of these indicators, interviewing with experts were carried out. The values of indicators were normalized based on TOPSIS and AECIEI methods. The normalized indicators values could be summed after applying the weight of each indicator to its value.
(3) Assessing results of scenarios by evaluating sustainable development indicators: the IA and all scenarios were modeled by AIMSUN software, and indicator’s values were done through AIMSUN results in an indirect and direct procedure.
(4) Ranking scenarios and choosing the great scenario. Combining indicators were done by the TOPSIS method. Ranking the scenarios was done by this method, and then the best scenario was chosen. The combined indicator was required to rank the scenarios, which could help to compare scenarios in a quick and easy manner [36,44].
(5) Defining a simplified approach. The AECIEI was defined based on the MCDA approach; by using this method, the effect of changes intensity of each indicator was applied in MCDA. Although the previous methods applied the weight of each indicator to sum the effect of all indicators for sorting and finding the best scenario, the effect of changes intensity was not applied on sustainability evaluation. When there were various values of an indicator in various scenarios, and difference of these values was significant, and these changes had a different effect on sustainability, AECIEI could apply the effect of changes intensity to the normalized value of each indicator in a scenario against the values of the same indicator in other scenarios. On the other hand, when there was a high change in the value of an indicator of a scenario against other scenarios, the effect of changes intensity should be applied. For example, the effect of air pollution or green space destruction in a different range was not the same on sustainability; therefore, they should be normalized based on the effect of changes intensity on sustainability. By interviewing with experts, the effect of changes intensity of each indicator could be specified. In this study, the results of AECIEI were compared with the result of TOPSIS.

### 3.2. Data

The interviewing with thoroughbred experts and precious information educed from reports of transportation and traffic organization of the Tehran Municipality (TTOTM) were used in this study. Twenty-four interviews were done with experts involving six employees of the subway and bus and bus organization, six employees of municipal transportation and urban-planning departments, six members of transportation and urban planning faculties, and six employees of the City Council in Tehran. The interviewed members were chosen from urban planners and transport experts who have an experience of more than 15 years in their specialty. For analyzing and evaluating scenarios, Trans CAD 5, AIMSUN 8.0, and MATLAB program were used. Trans CAD was used to perform the macroscopic survey; Trans CAD is a geographic information system (GIS) specifically designed for transportation professionals to demonstrate, analyze, and manage transportation studies and projects [45]. AIMSUN is a microscopic traffic simulator developed by transport simulation systems (TSS), dealing with arterials, ring road, highways, interchanges, and freeways [46]; in the present investigation, AIMSUN software was used for modeling micro-scale betterment scenarios and calculate the value of chosen indicators in an indirect and direct manner. To select the better scenario, TOPSIS and AECIEI methods were used. TOPSIS is an MCDA approach, which could help to specify a better scenario based on environmental, social, and economic selected indicators by considering the shortest and longest distance from the positive and negative ideal point, respectively. In this study, TOPSIS and AECIEI analyses were done by MATLAB software. 

### 3.3. Case Study

Tehran is the capital city of Iran; it is a city with 8.85 million inhabitants (year 2016) spreading over 730 square kilometers. In the metropolitan area of Teheran there is a large number of industrial activities, attracting a great amount of daily commuting from the neighboring cities such as Karaj, Shahriar, and Pardis.

**Figure 1:**
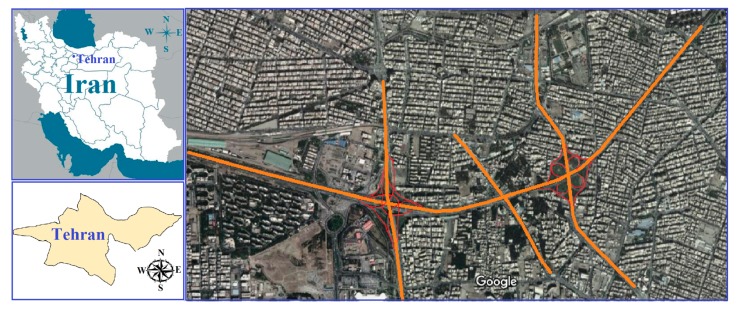
The interchange between Sheikhfazolah and Yadegar highways (Tehran, Iran).

The high growth rate of population and high economic blossoming in Tehran are a reason for ever-increasing demand transportation causing heavy traffic in the metropolitan area. Highways and interchange of highways play an important role to decrease traffic problem. There are four highways connecting north to south of Tehran; one of them is the Yadegar highway. Three highways connect Tehran with Karaj and other populated cities located west of Tehran; one of them is the Sheikhfazolah highway. As a consequence, the interchange between Sheikhfazolah and Yadegar highways plays an important role in the transportation of people who live or work in Karaj and other neighborhood cities located west of Tehran. Figure 1 shows the interchange located between Sheikhfazolah and Yadegar highways.

There are three lanes in each direction of Sheikhfazolah and Yadegar highways. Sheikhfazolah highway is a West-East route where 57,919 vehicles daily pass in a westerly direction and 87,784 in an easterly direction. Yadegar highway is a North-South route where 79,287 vehicles daily pass through south to north and 39,737 through north to south. Vehicles include taxis, cars, and buses representing the average daily traffic (ADT). According to the transportation demand from Karaj to Tehran, the congestion level of the interchange between Sheikhfazolah and Yadegar highways is rather high.

## 4. Study Framework

### 4.1. Scenario Building

Describing scenarios that lead to a sustainable transport system is a fundamental part of the decision-making process since it includes a high level of ambiguity about the future impacts of a transport infrastructure in the transportation and environment system [47]. Lots of investigators have approved to present sustainable transport scenarios, but it is challenging to evaluate all dimensions of sustainability by considering each scenario. According to field researches and interviews with experts, kind of system interchange (as cloverleaf interchange), high transportation demand, median divider island, and road surfacing weaving are four main transportation issues in Sheikhfazolah-Yadegar interchange, causing traffic congestion.

Specialists have been requested to present some solutions that could deal with the transportation issues in these interchange and highways. Therefore, the scenarios were defined according to the specialists’ suggestions by accounting for their expertise. Moreover, the appropriate advancement scenarios to the Sheikhfazolah-Yadegar interchange and its highways are elicited from advancement professional projects of TTOTM.

**Figure 2:**
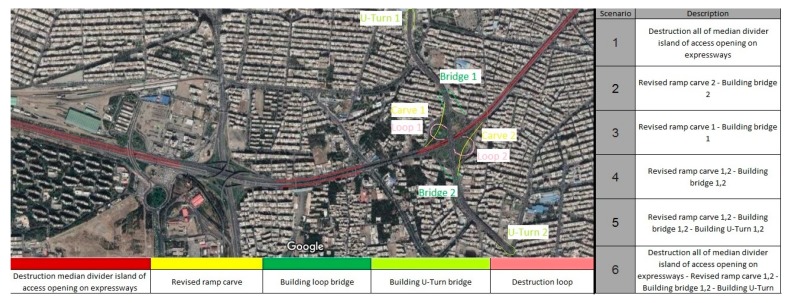
Six proposed scenarios.

Six feasible and acceptable scenarios are created based on the available and utilizable resources, as shown in Figure 2. The first and sixth scenarios improve network condition for private transportation; all of the scenarios except the first scenario improve road surfacing weaving in Sheikhfazolah-Yadegar interchange and its highways. The first scenario reduces traffic by eliminating the median divider island next to the expressways access from Sheikhfazolah highway. According to safety impact, the w-beam guardrail is considered in scenario 1 and 6 to improve safety because of the removal of the median divider island.

### 4.2. Specifying Influence Area (IA)

In this study, the same IA is chosen for all scenarios in order to obtain acceptable, reliable, and validated values of indicators. IA is determined by travel demand, saturation and free flow travel time, signal timing, and capacity, by using Trans CAD 5.0 software.

**Figure 3:**
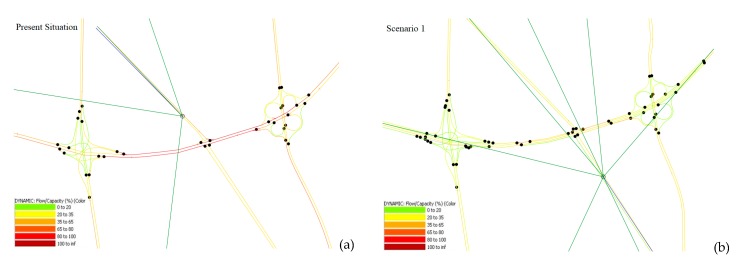

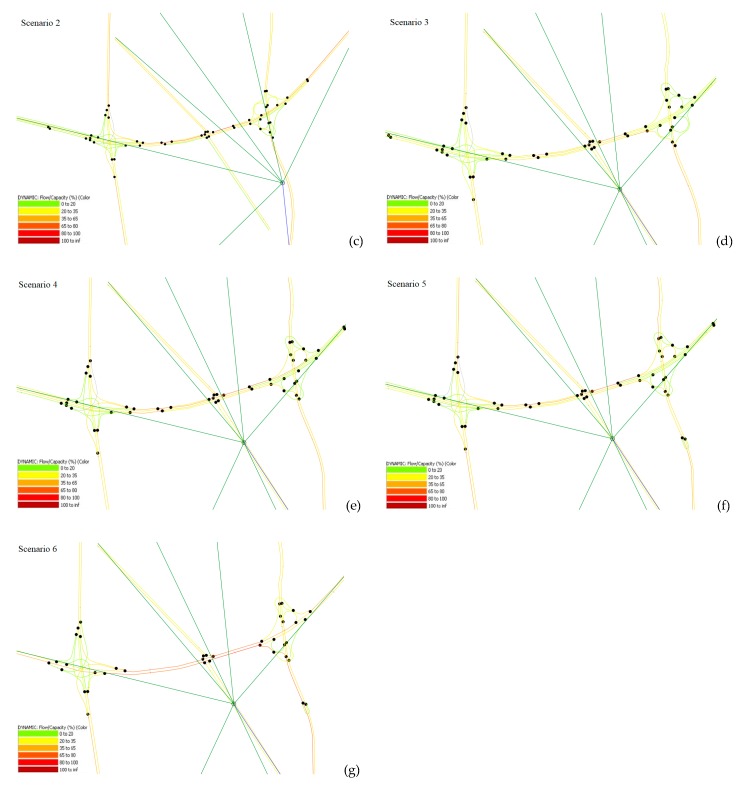
Simulation of the current transportation network and flow per capacity (F/C) ratio for the present situation (**a**), scenario 1 (**b**), scenario 2 (**c**), scenario 3 (**d**), scenario 4 (**e**), scenario 5 (**f**), and scenario 6 (**g**).

The six proposed scenarios were modeled by using AIMSUN software, and the traffic flow was estimated by traffic models. The flow per capacity (F/C) for all scenarios and the simulation of the transportation network in the current situation are shown as Figure 3.

Legend of AIMSUN results shows flow per capacity (F/C): the dark red, red, orange, light orange, yellow, and green color specify the limit value of F/C in the simulation models where the dark red and green show the highest and lowest traffic flow per capacity, respectively. A user equilibrium procedure was applied in this study, because this is the most widely utilized trip assignment procedure for car trips. In this method, travel times of each traveller cannot be improved by shifting routes [44].

**Figure 4:**
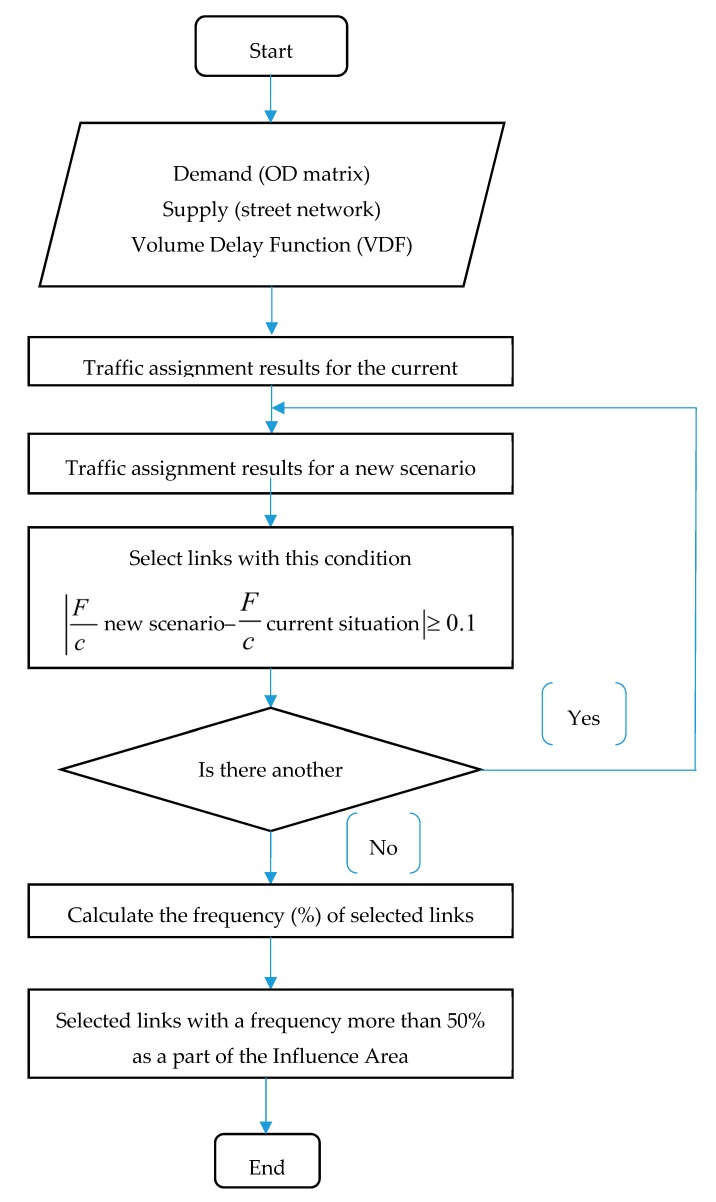
A flowchart of specifying influence area (IA) process.

According to Figure 3, it was specified how the proposed scenarios could improve the traffic condition on the network’s links. The links where the total value of the F/C difference between the current situation and each proposed scenario was more than 0.1 were chosen. This process was implemented repetitively for all proposed scenarios, and then, the choice frequency of each link was calculated; for example, if the links are chosen for six times the frequency is 100%. In the present investigation, the links with the choice frequency more than 50% was selected as a part of the IA. Figure 4 explains all the steps of this process in a flow chart. According to Figure 3, the extent of IA includes two street parts and two interchanges with their highways. These streets and interchanges are named Chobtarash, Teimori, Sheikhfazolah–Yadegar, and Sheikhfazolah–Jenah, respectively. Moreover, 4.4 km of the Sheikhfazolah highway, 2.2 km of the Yadegar highway, 2.2 km of the Jenah highway, 0.62 km of Chobtarash, and 0.7 km of Teimori were modeled as the extent of IA.

### 4.3. Choosing Eminent Sustainable Indicators

In the sustainable development concept, indicators can explain and demonstrate the status of each scenario in a specified index. On the other hand, indicators are quantitative and qualitative tools for comparing all scenarios with the present situation [48]. To select better sustainable transportation infrastructure, sustainable indicators for each scenario and the present situation should be calculated; they should be normalized and congregated for each scenario based on TOPSIS and AECIEI for comparing all scenarios with the present situation. In the present study, indicators of sustainable transportation were divided into three groups, where these groups include environmental, social, and economic indicators [49,50].

The sustainability evaluation of transport infrastructure needs to consider the environmental, social, and economic aspects of each scenario. The most important challenging issue in the sustainable evaluation of urban transportation is related to select and employ the appropriate indicators [14]. As a result, selecting and identifying effective sustainable transportation indicators is essential in each sustainability evaluation study. Although selecting a small set of indicators can simplify the evaluation, it causes overlooking the important impact. In contrast, selecting a large set of indicators improves the accuracy of the evaluation, but the cost of analyses is increased. Several criteria are employed to select appropriate indicators [13,16,17,18,21]:

1. There is a relationship between the selected indicators with environmental, economic, and social aspects;
2. The chosen indicators should be understandable and useable by anybody;
3. Users can understand how the final value is calculated;
4. The selected indicators should be predictable;
5. All selected indicators should be comparative;
6. The selected indicators should be calculated at the appropriate spatial and temporal scales;
7. The data of each selected indicator should be reliable and available at a reasonable cost;
8. The selected indicators should be measurable, quantifiable, and reproducible;
9. Each selected indicator should be independent of each other.

According to the above-mentioned criteria, the indicators of the present study are chosen (Table 1). Several transportation parameters, such as total travel time (TTT), vehicle kilometer travelled (VKT), speed changes, and length of the main road, were not considered directly as indicators, although they were applied in quantifying the selected indicators, like air and noise pollution and fuel consumption. The spatial scale restricted the number of the assessed indicators. For instance, traffic analysis zones (TAZs) can be employed to determine access to opportunities, like entertainment, services, and workplaces that are distributed spatially in the urban zone; therefore, this indicator is not appropriate to employ in the present study area.

### 4.4. Indicator Quantification

#### 4.4.1. Environmental Indicators

Environmental indicators considered in this study included air pollution, carbon dioxide emission, fuel consumption, green space destruction, and land consumed, as described in the following.

Air pollution. There are some models that can simulate air pollution emission of vehicles. In Iran, there are vehicles with various technologies, fleet age, and different fuel quality during a year; therefore, accurate air pollution emission models should be used for these vehicles and local situation. Although AIMSUN models can simulate the emission of air pollution for each vehicle type and traffic situation, the local models for calculating air pollution emission are more reliable. Ebrahimi and Ghanbari [51] proposed a non-linear equation to estimate air pollution emission by eight types of vehicles by improving air quality emission models for urban traffic in Iran. In addition, serious studies are carried out to simulate and estimate air pollution emission of vehicles in Iran by obtaining a more reliable estimation [52,53]. These models could estimate air pollution emission of each type of vehicles based on the average speed of them. In addition, based on vehicles speed in one kilometer, nitrogen oxides (NOx), hydrocarbon (HC), and carbon monoxide (CO) from buses and cars were calculated per grams; therefore, the value of air pollution emission in current situation and each scenario could be calculated in kilograms per hour based on the average vehicles speed.

Carbon dioxide emission. Two investigations were conducted to simulate and estimate carbon dioxide (CO_2_) emissions of vehicles in Iran [52,53]. Based on these studies, the CO_2_ emission for all scenarios and the present situation could be calculated. As above-mentioned, based on the vehicles speed in one kilometer CO_2_ from cars and buses were calculated per grams; hence, the value of CO_2_ in each scenario and current situation were calculated in kilogram per hour based on the average speed of vehicles.

Fuel consumption. Fossil fuel is non-renewable energy, and this energy is not sustainable. Two types of common fossil fuel include gasoline and diesel fuel, which are used by cars and buses in Iran respectively. Some studies are proposed several models to estimate fuel consumption in Iran where fuel consumption for each type of vehicle is calculated based on its average speed [52,53]. In the present study, the average speed of vehicles was derived from AIMSUN models; therefore, the average consumed fuel for each type of vehicle was calculated. Finally, based on average fuel consumption for each type of vehicle and number of vehicle type, total volumes of fuel consumption per hour were calculated for each scenario separately.

Green space destruction and land consumed. These parameters are significant issues in environmental evaluation. When transportation infrastructure is developed, the area of green space is decreased, and the areas of land consumed are increased. The values of green space and land consumed were determined by comparing each scenario with the current situation.

#### 4.4.2. Social Indicators

Social indicators considered in this study included safety, noise pollution, and average travel time, as described in the follow.

Safety. In recent years, regression models have been used to forecast all types of crashes based on the Highway Safety Manual (HSM). In the present investigation, HSM is developed to forecast the values of crashes without regard to collision type. The analyzed study area includes two interchanges, two highways, and two urban streets. In this study, a safety performance function is used to evaluate the safety indicator, which is a regression model predicting the average crash frequency based on geometric plan and traffic control specifications referred to our case study [54,55,56]. Accident modification factors (AMFs) are used to regulate the estimated safety indicator from the safety performance function. For predicting crashes in a region outside the U.S., the local condition should be applied to HSM algorithm [55]. Driver training, population, meteorological condition, and geographic specifications are effective variables causing the difference value between observed and forecasted crash frequencies in different condition [57]. In this study, the accident models presented by D’Agostino [57] were used to predict crashes in each scenario. To calibrate these models, calibration factors were determined by comparing the observed and forecasted total number of crashes in a sample dataset. To achieve this aim, crashes reported by the traffic police centre of Tehran in the interchanges, highways, and streets of the current situation were compared with actual crashes. For highways and interchanges, the calibration factors C_r_ and C_i_ were respectively determined by obtaining the values 1.57 and 1.43. The correlation coefficient (R^2^) between observed and forecasted crash values were close to 0.78 and 0.81, respectively. These values of R^2^ show the predicted values were acceptable. Definitively, the crash frequency was forecasted for all interchanges, highways, and streets in six proposed scenarios and current situation as the safety indicator.

Exposure to the noise level above 55 dB. Road traffic can produce excessive noise, which causes psychological stress, sleep disorder, displeasure, and offense [58]. Some traffic noise forecasting models have been presented in recent years. For example, Givargis and Mahmoodi [59] determined sound pressure levels in Iranian roads by converting and developing the UK calculation of road traffic noise (CORTN). Based on the reports of Iran’s Council of Ministers in 2000, the land use in the region of the analyzed interchange is prevalently residential; therefore, the noise level should not be less than 55 and 45 dB in during daytime and night-time respectively. These values for silent zones should be less than 50 and 40 in during daytime and night-time respectively. According to the highest limit of noise levels for a residential zone, exposure to the noise level above 55 dB should be determined for all proposed scenarios and current situation. To achieve this goal, the algorithm proposed by Givargis and Mahmoodi [59] was used to calculate a distance from highways and streets with this noise level.

Average travel time. When traffic density increases, average travel time increases too; therefore, public satisfaction and system reliability decrease. As a result, average travel time was one of the social indicators. Combination of bus and car travel time was used as the travel time indicator in the present work. Lower and higher values of travel time demonstrate more and less efficient network, respectively. By AIMSUN we could calculate travel time values of buses and cars. Equation (1) was utilized to determine average travel time, which is formulated as:

(1) $$ATT=\frac{\left(TTT_{car}\times Avg.CarOccupancy)+(TTT_{bus}*Avg.BusOccupancy\right)}{\left(V_{car}\times Avg.CarOccupancy)+(V_{bus}*Avg.BusOccupancy\right)},$$

where *TTT_bus_*, *TTT_car_*, and *ATT* are total travel time spent by buses and cars and average travel time. *V_bus_* and *V_car_* are the total volumes of buses and cars. The average bus and car occupancy were selected 20 and 1.7 respectively, based on the previous studies in Iran [13,23,60].

#### 4.4.3. Economic Indicators

Economic indicators considered in this study included capital costs, and maintenance and repair costs, as described in the follow.

Capital costs. According to values of consumed lands and construction price, the capital costs were calculated for each proposed scenario. Based on material and construction price in Iran [61], we established the land price of the studied region is $1000 (US dollars/m^2^), and $1500 for commercial and residential land, respectively; in addition, the construction cost (US dollar per m^2^) for building a bridge, building a U-turn bridge, revising a ramp curve, and destruction of a median strip is $636, $668, $19, and $22, respectively. Building a U-turn bridge and building a bridge have different prices because of the destruction of houses, which are located beside highways. Revising a ramp curve and destruction of a median strip have a different capital cost because of the destruction asphalts in revising ramp curve [62]. In this study, the limitation regarding the budget was 1.2 million dollars.

Maintenance and repair costs. To estimate maintenance and repair price for each scenario, determining road assets were required. As the Economic Commission for Latin America and Caribbean (ECLAC) proposes, the maintenance and repair price is between 2.5% and 3.5% of a road network’s asset [63]. In the present investigation, 2.5% of the amount of the assets was considered to calculate maintenance and repair cost based on expert opinions.

### 4.5. Sorting and Ranking Based on TOPSIS

For comparing the proposed scenarios, various aspects should be matched. CBA is an appropriate tool to compare costs and benefits from proposed scenarios. Decreasing crashes and time saving can be defined as user benefits, whereas a negative effect such as using non-renewable resources and costs can be applied. However, a quantitative evaluation of some indicators such as green spaces destruction, exposure to the noise level, and air pollution could introduce certain problems [64,65]. As a consequence, many authors used MCDA to evaluate scenarios in order to make a decision among more scenarios. According to an integrated sustainable evaluation, this method can demonstrate the best option between all scenarios [23,66,67]. The method is based on specifying and determining the amount of some sustainable indicators. According to the relative effect of each indicator, the weight of each indicator is chosen, based on the suggestions from interviewing with experts. Finally, the scenarios are sorted by TOPSIS method [22,26]. TOPSIS is easy to utilize, and it can present more integrated data by combining sub-indices into one overall index. It can explicate all scenarios simply. In the present investigation, the TOPSIS approach was used to evaluate sustainability, and it includes some steps as in the following.

#### 4.5.1. Indicator Normalization

TOPSIS can determine the lowest and highest distance from an ideal point, which demonstrates the best and worst alternative, respectively. First of all, a matrix n * m (*A*) should be created, which includes m alternatives and n indicators. Each alternative is an option, which should be evaluated for making a decision. Indicators can demonstrate a feature of each alternative, which is determined. On the other hand, indicators can convert the qualitative feature to values for evaluating and comparing each scenario; therefore, this method can compare and evaluate the quantitative and qualitative feature of scenarios. To normalize the information, a matrix should be calculated. Matrix *A[a_ij_]* and the elements of matrix *R[r_ij_]* are shown in Formulas (2) and (3).

(2) $$A_{ij}=\left[\begin{array}{cccc} a_{11} & a_{12} & \ldots & a_{1n}\\ a_{21} & a_{22} & \ldots & a_{2n}\\ & & \begin{array}{l} .\\ .\\ . \end{array} & \\ a_{m1} & a_{m2} & \ldots & a_{mn} \end{array}\right].$$

(3) $$r_{ij}=\frac{a_{ij}}{\sqrt{\sum_{k=1}^{m}a_{kj}{}^{2}}}.$$

#### 4.5.2. Selecting Weights of Indicators

A weight is assigned to each indicator according to its importance in sustainability evaluation: the greater the effect on sustainability is, the greater the weight is. Summation of the weight of all indicators must reach 1. Matrix *V [v_ij_]* is calculated based on applying weight matrix into the matrix *R*, as formulated in (4).

(4) $$V_{ij}=\left[\begin{array}{cccc} w_{1}r_{11} & w_{2}r_{12} & \ldots & w_{n}r_{1n}\\ w_{1}r_{21} & w_{2}r_{22} & \ldots & w_{n}r_{2n}\\ & & \begin{array}{l} .\\ .\\ . \end{array} & \\ w_{1}r_{m1} & w_{2}r_{m2} & \ldots & w_{n}r_{mn} \end{array}\right].$$

Then, the distance between alternative *i* and the ideal alternative should be calculated (A*), and the distance between alternative *i* and the worst alternative (*A^−^*), as reported in Formulas (5)–(8).

(5) $$A^{*}=\{(\max v_{ij}|j\in J),(\min v_{ij}|j\in J)\}.$$

(6) $$A^{*}=\{v_{1}{}^{*},v_{2}{}^{*},\ldots ,v_{n}{}^{*}\}.$$

(7) $$A^{-}=\{(\min v_{ij}|j\in J),(\max v_{ij}|j\in J)\}.$$

(8) $$A^{-}=\{v_{1}{}^{-},v_{2}{}^{*},\ldots ,v_{n}{}^{-}\}.$$

Transportation purposes of Tehran at 2025 horizon are selected based on interviews with experts, as shown in Table 2. The important goals include decreasing traffic congestion, safety promotion, decreasing air pollution and noise pollution, improving the visual aesthetics, revising reasons of weaving, increasing speed and fluidity of traffic, creating easy access in highways and streets.

Ten forms were used to calculate the weight of all selected indicators and the effect value of changes intensity for each indicator. The weight of each indicator was calculated by the Expert Choice program. The ten forms include personal information of each expert, questions for determining the effect value of changes intensity, weights of indicators, and the proposed scenarios.

**Figure 5:**
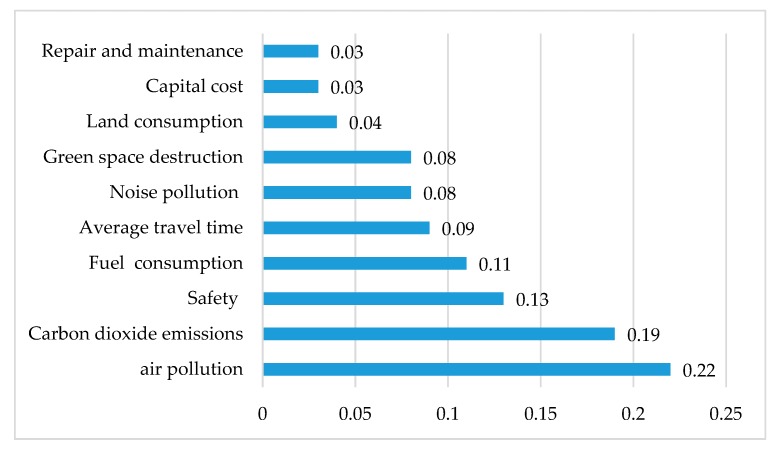
The weight of sustainable indicators based on the expert’s point-of-view (overall inconsistency 0.06).

Figure 5 shows the weight of all selected sustainable indicators. Based on experts’ opinion, the weight of air pollution has the greatest value (0.22) among the other indicators because air pollution is the major problem of highly populated cities in Iran such as Tehran. The second most important indicator is carbon dioxide emission that causes climate change (0.19), whereas the third one is safety, with a value of about 0.13. The value of fuel consumption weight is 0.11 because of an international crisis in recent years regarding non-renewable resources.

Green space destruction plays an important role to decrease air pollution and CO_2_ concentration; therefore, it has an important effect on public health. As a consequence, its value was 0.08 in the present study. The weight of average travel time, noise pollution, and land consumption were 0.09, 0.08, and 0.04 respectively. Economic indicators, which include maintenance and capital costs, have the lowest importance among all indicators (0.03). The summation of the weight of all indicators is 1.

#### 4.5.3. Finding the Best and Worst Solution Based on TOPSIS

Hwang and Yoon in 1981 presented the technique for order of preference by similarity to ideal solution (TOPSIS). This approach, which is a useful tool to find the best alternative against some alternatives, is one of the MCDA methods [68]. The method was completed by Yoon in 1987 and Hwang, Lai, and Liu in 1993 [69,70] (Yoon, 1987; Hwang et al., 1993). Criteria of distance for the ideal (s_i_*) and the worst (*s_i_^−^*) alternatives are calculated by Equations (9) and (10). To specify the best and worst alternatives, c_i_* should be calculated based on Equation (11).

(9) $$s_{i}^{*}=\sqrt{\sum_{j=1}^{n}{\left(v_{ij}-v_{j}^{*}\right)}^{2}}.$$

(10) $$s_{i}^{-}=\sqrt{\sum_{j=1}^{n}{\left(v_{ij}-v_{j}^{-}\right)}^{2}}.$$

(11) $$c_{i}^{*}=\frac{s_{i}^{-}}{s_{i}^{-}+s_{i}^{*}}.$$

### 4.6. Sorting and Ranking Based on AECIEI

In the AECIEI method, experts specify the effect of changes intensity; they select the effect of changes intensity from one to five, where 5 refers to changes having a very high effect and 1 a slight effect. Table 3 shows the value of each kind of effect based on AECIEI approach.

The effect value (*EV*) of changes intensity is used to normalize the values of indicators. As a result, the normalization of each indicator in AECIEI is carried out based on Equation (12).

(12) $$NI_{ij}=I_{ij}^{EV}/\sum I_{ij}^{EV}(i=1,2,\ldots ,n;EV=1,2,\ldots ,5),$$

where *I_ij_* is indicator *i* in scenario *j* among *n* scenarios and *m* indicators.

The weight of each indicator can be determined by interviewing with 24 experts or more. In this approach, the interviewed members should be selected from all urban planners and transport experts with an experience of more than 15 years in their specialty. Definitively, the weight of all indicators is calculated like the TOPSIS method; therefore, the weight value of each indicator in TOPSIS and AECIEI is the same. After normalizing the value of each indicator based on AECIEI, the weight of each indicator should be applied, and then, these values can be summed to determine the sustainability value (*SV*). In other words, *SV* of scenario *j* is calculated by Equation (13).

(13) $$SV_{j}=\sum_{i=1}^{m}\pm W_{i}\times NI_{ij},$$

where *N_ij_* is normalized value of indicator *i* in scenario *j* based on Equation (12), and *m* is the number of indicators in each scenario. The negative and positive effect of each scenario is applied by minus and plus as shown in Equation (13). *W_i_* is the weight value of each indicator, which is specified based on interviews by experts.

## 5. Results

### 5.1. Ranking Based on TOPSIS

Based on interviews with experts, intensive observation, and development infrastructure related to the case study, six proposed scenarios are presented to improve traffic condition of Sheikhfazolah-Yadegar interchange in Tehran. Table 4 shows the value of c_i_* for each scenario and current situation, the values of indicators, the normalized indicators values, and the weight for each indicator. According to Table 4, total hourly air pollution emissions of the do-nothing scenario (current situation) were 16.85 kg/h, while this parameter decreased in other scenarios. As a result, air quality improved in other scenarios; the total hourly air pollution emissions of scenario 6 were 11.93, approximately 30% less than the current situation. The second best alternative was scenario 1, where hourly emissions were 12.29, 27% less than the present situation. Total hourly carbon dioxide emissions of the do-noting scenario were 193.13 kg/h. In this case, the parameter had also improved in other scenarios: Carbon dioxide emissions decreased to 146.89 kg/h and 149.81 kg/h in scenarios 6 and 1 respectively; therefore, the value of this parameter decreased 23.9% and 22.4% in scenarios 6 and 1 against the value of current situation. According to changing system interchange in six scenarios, fuel consumption decreased. Fuel consumption was 4275.59 L/h in the current situation, and the value of this parameter was 3233.16 L/h and 3338.46 L/h in scenarios 1 and 6 respectively; thus, fuel consumption decreased 24.4% and 21.9% in scenarios 1 and 6 respectively, where scenario 1 was the best scenario in the evaluation of fuel consumption. The greenest space destruction related to scenarios 1 and 6 because all median divider islands on access opening in Sheikhfazolah highway were destructed. There was no green space destruction in other scenarios. More, in the current situation land consumption value was zero, and most land consumption relates to scenario 1 and 6. There were three median divider islands in Sheikhfazolah highway: one of them was located between north and south route; others were located in beside expressways for dividing local access. The median strips between expressways and local access were destruction in scenarios 1 and 6; therefore 28,760 m^2^ of green space were destruction in these scenarios. Based on green space destruction and land consumption, scenarios 1 and 6 were the worst scenarios. According to building a loop bridge in scenario 6, the land consumption in scenario 6 was more than scenario 1; therefore, the value of land consumption in scenarios 1 and 6 were 28,760 m^2^ and 29,320 m^2^ respectively.

Social equity is divided into safety, noise pollution, and average travel time per capita. The safety of all scenarios was the same approximately. The harmful effect of noise pollution is another indicator, evaluated in each scenario based on exposure to the noise level above 55 dB. The value of this parameter increased by 5.0% and 2.3% in scenarios 1 and 6 respectively. Average travel time per capita decreased in all proposed scenarios; specifically, it decreased by 17.92% and 16.50% in scenarios 1 and 6, respectively. Other scenarios decreased average travel time less than 14% by a comparison with the do-nothing scenario; thus, other scenarios improved travel time as well, but not as many as scenarios 1 and 6. Construction cost of transport infrastructure of scenarios 5 and 6 was 0.65 and 1.18 million US dollars, the higher capital cost of each scenario because the most loop bridge should be built in these scenarios. According to repair and maintenance costs, scenarios 1 and 6 were the costliest because more asphalt was required to repair in scenarios 1 and 6 against other scenarios. On the other hand, paved areas in scenarios 1 and 6 were more than other scenarios; therefore, more areas of asphalt were needed to repair yearly in these scenarios.

Based on a sustainable development index and the *c_i_* results, scenario 6 with *c_i_* equal to 0.653 was the best option in our study. Scenario 6 achieved the best score in three sustainable development indicators such as air pollution, CO_2_ emission, and safety, and it achieved the second score in average travel time and fuel consumption. Scenario 1, with a *c_i_* equal to 0.638 was the second best alternative based on sustainable development aims. Scenario 1 achieved the best score in average travel time, safety, and fuel consumption indicators and the second best score in two sustainable development indicators involving air pollution and CO_2_ emission. The do-nothing scenario ranked in third place with a *c_i_* equal to 0.349. According to the values of *c_i_* for each scenario and current situation, the worst scenario referred to scenario 5, because *c_i_* value was less than the values of other scenarios. Average travel time in scenario 5 was 354.78 s per person, which was the second worst value among the value of average travel time in all other scenarios and current situation. Scenarios 2, 3, and 4 improve air pollution, CO_2_ emission, fuel consumption, and average travel time in comparing with the current situation.

### 5.2. Ranking Based on AECIEI

Based on the previous studies, the different approach of MCDA demonstrated various results in different case studies [71,72]. In this perspective, applying the effect of changes intensity of indicator values can demonstrate different results against TOPSIS. To apply the effect of changes intensity of indicator values, AECIEI was introduced and used for the first time in the present investigation. Table 5 shows the results of AECIEI method; hence, the results of TOPSIS and AECIEI were compared. Effect value (EV) on changes intensity calculated based on interviews with experts was also reported in Table 5. EV of air pollution and carbon dioxide emissions was five, and it means that changes intensity in these indicators had a very high effect on sustainability. According to Table 5, changes intensity of fuel consumption and safety had a high effect on sustainability; therefore, EV of these indicators is four. The effect of changes intensity of green space destruction, average travel time, and noise pollution could be applied to the moderate effect on sustainability and EV of these indicators was determined as three. EV of other indicators was calculated as one. On the other hand, the effect of changes intensity of land consumption, capital cost, and repair and maintenance was the slight effect on sustainability.

By adopting AECIEI approach, the best option was the scenario 5 because this scenario had the minimum negative effect on sustainability. Scenario 3 and 2 with *SV_i_* equal to −0.132 and −0.143 were the second and third best option with respect to AECIEI results, respectively. Based on the results of Table 5, the do-nothing scenario with a *SV_i_* of −0.177 shows the maximum negative effect on sustainability; therefore, this scenario was the worst option. Although scenario 6 was the best option with respect to TOPSIS results, this scenario with *SV_i_* equal to −0.149 was the second worst scenario based on AECIEI approach; scenario 1, with *SV_i_* equal to −0.144 was the third worst option. There was 28,760 m^2^ green space destruction in scenarios 6 and 1. In addition, based on results of noise pollution and repair and maintenance results indicators, scenarios 1 and 6 achieve the first and second worst results in comparing with other scenarios; therefore, it seems that they should not be selected as the best scenarios. On the other hand, it seems that the best option, which was selected by TOPSIS, was not accurate.

Definitively, results of Table 4 and Table 5 show that ranking scenarios in AECIEI were not similar to TOPSIS. All scenarios were sorted based on the negative effect in AECIEI method; for example, the negative effect of the current situation was more than other scenarios. The results show that scenario 5 caused minimum negative sustainability effect; therefore, scenario 5 was the best option with respect to AECIEI method. In addition, although there was 28,760 m^2^ green space destruction in scenarios 1 and 6, there was no green space destruction in other scenarios. The green space could improve air pollution and climate change; therefore, this indicator had a significant effect on sustainability evaluation. This reason explains why scenarios 6 and 1 were not the best options, although, they were selected as the first and second best options based on the results of TOPSIS. According to the results of this study, the AECIEI method could consider the local situation to sort scenarios better than other methods by applying the effect of changes intensity on sustainability in mega cities where there were some environmental and social problems.

The current system interchange of Sheikhfazolah-Yadegar is a Cloverleaf interchange, because this interchange should be used in low populated areas. According to transportation demand in this region, changing system interchange can improve traffic condition. Based on the AECIEI approach, sustainable development index shows that scenario 5 could improve sustainability in this interchange, although this scenario was the worst option based on the TOPSIS method; moreover, scenario 6 was the best option based on the TOPSIS method, although this scenario was the second worst option based on the AECIEI method. According to the results, TOPSIS method focused on the weight of each indicator and value of each indicator to find the best scenario. On the other hand, there were some sensitive environmental and social problems in mega cities, and increasing the value of them cause essential problems on sustainability. AECIEI could consider the effect of changes intensity in all scenarios; therefore, this method was introduced and proposed in sustainability evaluation for mega cities.

## 6. Conclusions

There were some challenges towards sustainable societies to achieve sustainable development plan in developed and developing countries, where the feasible and cost-effective procedures were essential to solve this kind of problem. In the present study, sustainable development analysis on Sheikhfazolah-Yadegar interchange was carried out to specify the best alternative transportation infrastructure. Sustainable development indicators at micro-scale were selected to evaluate proposed scenarios. The system interchange of Sheikhfazolah-Yadegar is a Cloverleaf interchange, being the low building cost the major advantage of this type of interchange, and the increasing road surfacing weaving the major problem; therefore, this interchange was used in a low population density city. According to the system interchange and results of interviews and observations, the system interchange of Sheikhfazolah-Yadegar should be changed because Tehran is a crowded population area with an increasing transportation demand. As a result, many vehicles should utilize this interchange to reach Tehran from Karaj and other neighborhood towns to Tehran. As a consequence, the system of this interchange plays an important role to improve traffic condition; therefore, changing the system interchange in this area could improve environmental, social, and economic condition. To achieve this aim, six proposed scenarios were analyzed. To compare these scenarios, a similar study area was selected, and Trans CAD 5.0 software was used to determine the IA of scenarios.

According to environmental and social problems growth in Tehran and highlighted in 2025 perspective, some indicators were selected in this study, which could cover all criteria of sustainability. To find the best scenario with respect to environmental, social, and economic indicators, the MCDA method was used and the TOPSIS and AECIEI methods were selected to find the best alternative from all scenarios and the current situation. AECIEI is an MCDA approach that can apply the effect of changes intensity of indicator values on sustainability based on the effect value of changes intensity. AECIEI shows different results by comparing with TOPSIS. Scenario 5 was selected the best scenario based on the AECIEI method, although scenario 6 was the best scenario based on TOPSIS. There was no green space destruction in scenario 5, although there was 28,760 m^2^ green space destruction area in scenario 6. According to environmental problems in mega cities such as low green space area, it seems that the AECIEI method was more successful than the TOPSIS approach for finding the best scenario.

Based on the TOPSIS method, in order to compare the current situation and proposed scenarios the *c_i_* amounts were used, which results show that changing the system interchange from Cloverleaf interchange to improved interchange was essential. Related to sustainable development index, scenario 6 was the best option based on TOPSIS because of the low distance from this scenario to the ideal point. In the Middle East, the cost of fuel was cheaper than other countries; therefore, there was a dependency on private transportations in the cities located in the Middle East, such as Tehran. Although increasing fuel cost and improving public transportation can help to improve the transportation problem, improving system interchange in crowded cities such as Tehran is also required.

According to AECIEI approach, *SV_i_* amount should be calculated to find the best option, and this parameter shows the negative effect of each scenario on sustainability. The results show that the current situation was the worst scenario; therefore, six alternative options were offered by experts to improve Sheikhfazolah-Yadegar interchange. Based on AECIEI results, scenarios 5 and 3 were the first and second best options respectively, having the minimum negative effect on sustainability. The AECIEI approach normalized the value of each indicator based on the effect of changes intensity of each indicator on sustainability. Based on a different method for normalizing indictors, AECIEI demonstrates different results in comparing with another MCDA method (like TOPSIS in this investigation). Definitively, by considering sensitive environmental and social problems in crowded cities, AECIEI could sort alternatives and select the best option better than the TOPSIS method.

Despite the innovative aspects introduced, this work had limitations because of the restrict number of considered sustainable transportation indicators. It depended on the unavailability of certain data in Tehran, as fatalities and injuries in traffic crashes, proportion of residents, fuel price, point to point travel cost, total vehicle-miles travelled, total time spent in traffic, and user welfare changes. Future developments of this works will be focused on the enrichment of more transportation parameters.

## Figures and Tables

**Table 1:** Chosen sustainable transportation indicators.

Sustainability Dimension	Area	Indicator	Unit
Environmental indicators	Air pollution	NO_x_, HC and CO emission per hour	kg/h
	GHG emission	CO_2_ emissions per hour	kg/h
	Consumption of natural resources	Fuel consumption	L/h
		Green spaces destruction	m²
		Land consumed for transport	m²
Social indicators	Safety	Average crash frequency	accident/km
	Public satisfaction	Average travel time	second/person
	Noise pollution	Exposure to noise level above 55 dB	m²
Economic indicators	Operator costs	Capital cost	dollar
		Maintenance and repair cost	dollar

**Table 2:** Transportation purposes of Tehran at 2025 horizon.

Area	Goals
Interchanges, Highways, and relevant streets	-Decreasing traffic congestion
	-Safety promotion
	-Decreasing air pollution, CO_2_ emission and noise pollution
	-Improving the visual aesthetics
	-Revising reasons for weaving
	-Increasing speed and fluidity of traffic
	-Creating easy access to highways and streets

**Table 3:** Classification of each effect value.

Kind of Effect	Effect Value (EV)
Very high effect	5
High effect	4
Moderate effect	3
Low effect	2
Slight effect	1

**Table 4:** Values of indicators and technique for order of preference by similarity to ideal solution (TOPSIS) outputs.

Indicator	Environmental Indicators					Social Indicators				Economic Indicators	c_i_*
	Air Pollution	Carbon Dioxide Emissions	Fuel Consumption	Green Space Destruction	Land Consumption	Safety	Average Travel Time per Capita	Exposure to Noise Level above 55 dB	Capital Cost	Maintenance and Repair	
	(kg/h)	(kg/h)	(L/h)	(m²)	(m²)	(acc/year)	(s/pers)	(m²)	(Million $)	(1000 $)	
Weight	0.22	0.19	0.11	0.08	0.04	0.13	0.09	0.08	0.03	0.03	
current Sc.	16.85	193.13	4275.6	0.00	0.00	405.44	358.45	553,151.4	0.00	1071.6	0.35
	0.43	0.43	0.43	0.00	0.00	0.38	0.41	0.37	0.00	0.27	
Scenario 1	12.29	149.81	3233.2	28,760	28,760.0	405.41	294.20	580,887.4	0.53	2222.0	0.64
	0.32	0.32	0.32	0.71	0.70	0.38	0.34	0.39	0.35	0.56	
Scenario 2	14.83	191.13	4248.1	0.00	290.0	405.44	308.54	553,772.9	0.20	1072.0	0.29
	0.38	0.42	0.42	0.00	0.01	0.38	0.36	0.37	0.13	0.27	
Scenario 3	16.18	167.48	3716.0	0.00	270.0	405.44	335.98	553,608.2	0.20	1072.8	0.28
	0.42	0.37	0.37	0.00	0.01	0.38	0.39	0.37	0.13	0.27	
Scenario 4	16.38	176.77	3927.7	0.00	560.0	405.44	339.15	553,395.2	0.39	1073.2	0.32
	0.42	0.39	0.39	0.00	0.01	0.38	0.39	0.37	0.26	0.27	
Scenario 5	13.18	165.83	3670.9	0.00	560.0	405.44	354.78	552,769.4	0.65	1089.2	0.21
	0.34	0.37	0.37	0.00	0.01	0.38	0.41	0.37	0.43	0.27	
Scenario 6	11.93	146.89	3338.5	28,760	29,320.0	405.41	299.30	565,795.7	1.18	2239.6	0.65
	0.31	0.32	0.33	0.71	0.71	0.38	0.34	0.38	0.77	0.56	

**Table 5:** Values of indicators and applied effect of changes intensity in each indicator (AECIEI) outputs.

Indicator	Environmental Indicators					Social Indicators			Economic Indicators		*SV_i_*
	Air Pollution	Carbon Dioxide Emissions	Fuel Consumption	Green Space Destruction	Land Consumption	Safety	Average Travel Time per Capita	Exposure to Noise Level above 55 dB	Capital Cost	Maintenance and Repair	
	(Kg/h)	(Kg/h)	(L/h)	(m²)	(m²)	(acc/year)	(s/pers)	(m²)	(Million $)	(1000 $)	
Weight	0.22	0.19	0.11	0.08	0.04	0.13	0.09	0.08	0.03	0.03	
Effect Value	5	5	4	3	1	4	3	3	1	1	
current Sc.	16.85	193.13	4275.6	0.00	0.00	405.44	358.45	553,151.4	0.00	1071.6	−0.177
	0.26	0.24	0.22	0.00	0.00	0.14	0.18	0.14	0.00	0.11	
Scenario 1	12.29	149.81	3233.2	28,760	28,760.0	405.41	294.20	580,887.4	0.53	2222.0	−0.144
	0.05	0.07	0.07	0.50	0.48	0.14	0.10	0.16	0.17	0.23	
Scenario 2	14.83	191.13	4248.1	0.00	290.0	405.44	308.54	553,772.9	0.20	1072.0	−0.143
	0.14	0.23	0.22	0.00	0.00	0.14	0.12	0.14	0.06	0.11	
Scenario 3	16.18	167.48	3716.0	0.00	270.0	405.44	335.98	553,608.2	0.20	1072.8	−0.132
	0.21	0.12	0.13	0.00	0.00	0.14	0.15	0.14	0.06	0.11	
Scenario 4	16.38	176.77	3927.7	0.00	560.0	405.44	339.15	553,395.2	0.39	1073.2	−0.147
	0.22	0.16	0.16	0.00	0.01	0.14	0.16	0.14	0.12	0.11	
Scenario 5	13.18	165.83	3670.9	0.00	560.0	405.44	354.78	552,769.4	0.65	1089.2	−0.107
	0.08	0.11	0.12	0.00	0.01	0.14	0.18	0.14	0.21	0.11	
Scenario 6	11.93	146.89	3338.5	28,760	29,320.0	405.41	299.30	565,795.7	1.18	2239.6	−0.149
	0.05	0.06	0.08	0.50	0.49	0.14	0.11	0.15	0.38	0.23	
